# Immunological and virological discordance among people living with HIV on highly active antiretroviral therapy in Tigray, Northern Ethiopia

**DOI:** 10.1186/s12879-021-06206-4

**Published:** 2021-06-12

**Authors:** Genet Gebrehiwet Hailu, Araya Gebreyesus Wasihun

**Affiliations:** Department of Medical Microbiology and Immunology, College of Health Sciences, Mekelle University, Mek’ele, Tigray Ethiopia

**Keywords:** HIV, Immunological discordance, Virological discordance, HAART discordant responses, Northern Ethiopia

## Abstract

**Background:**

People living with human immunodeficiency virus (HIV) with immuno-virological discordant responses are at an increased risk to develop acquired immunodeficiency syndrome (AIDS) and severe non AIDS events which are risk factors for death. This study was aimed to assess prevalence of immuno- virological discordant responses and associated risk factors among highly active antiretroviral therapy (HAART) users in Tigray, Northern Ethiopia.

**Methods:**

A cross sectional study was conducted from September to December 30, 2016 on 260 people living with HIV who started first line HAART from January 2008 to March 2016 at Mekelle hospital and Ayder comprehensive specialized hospital. Baseline and follow-up clinical data and CD4+ result were collected from patient charts. Besides, socio-demographic data and blood samples for CD4 _+_ count and viral load measurement were collected during data collection period. Fisher’s exact test, bivariate and multivariate logistic regressions were used for data analysis. *P*-value < 0.05 with 95% CI was considered as statistically significant.

**Result:**

Among the 260 study participants, 8.80% (95% Confidence Interval (CI) =8.77–8.84%) and 2.70% (95% CI = 2.68–2.72%) had virological and immunological discordant responses, respectively with an overall immuno-virological discordance response of 11.50% (95% CI = 11.46–11.54%). The median age of the study participants at HAART initiation was 35 (IQR: 28–44 years). More than half (58.1%) of the study participants were females. Age at or below 35 years old at HAART initiation (AOR ((95% CI) = 4.25(1.48–12.23), *p* = 0.007)), male gender ((Adjusted Odds Ratio (AOR) (95% CI) =1.71(1.13–1.10), *p* = 0.029)), type of regimen given ((AOR(95% CI) = 0.30 (0.10–0.88), *p* = 0.028)) and good treatment adherence ((AOR (95% CI) = 0.12 (0.030–0.0.48), *p* = 0.003)) were associated risk factors for virological discordant response. Likewise, immunological discordant response was associated with tuberculosis co-infections (*p* = 0.016), hepatitis B virus co-infections (*p* = 0.05) and low CD4+ count (≤100 cells/μl) at baseline (*p* = 0.026).

**Conclusions:**

Over all, immuno-virological discordance response was 11.5% in the study area. Males, low baseline CD4+ count, poor/fair treatment adherence, and TB and HBV co-infections were significantly associated with higher immuno-virological discordance. We recommend that decision of patient treatment outcome, regimen change and patient management response should be done using trends of both viral load and CD4+ count concurrently.

**Supplementary Information:**

The online version contains supplementary material available at 10.1186/s12879-021-06206-4.

## Background

The use of highly active antiretroviral therapy (HAART) has substantially improved the survival of people living with human immunodeficiency virus (HIV) by suppressing the viral load to undetectable levels and providing a consistent increase in the number of T lymphocytes both of which lead to slow progression of the infection towards AIDS [1–3].

Although, the goal of HAART is to suppress viral load to undetectable level and restore CD4+ count [4], there are groups of people living with HIV with immuno-virological discordant responses i.e. suppressed viral load to the undetectable level but still with poor immunological recovery (immunological failure) or good immunological response with detectable viral load (virological failure: viral load >1000copies/mL) [5, 6].

Studies on immuno-virological discordant responses among people living with HIV have reported from 29 to 33% [5, 7]. Immunological discordance among people living with HIV have been reported from 13.59 to 29% [5–13]. Factors such as being younger age [6], poor HIV treatment adherence, being male gender, lower baseline CD4+ count [2, 7, 9, 14], not taking treatments from three antiretroviral classes [15], and Hepatitis C co-infection [10] have been reported to increase risk of immunological discordant responses. In other hand, virological discordant response has also reported from 5 to 17% [5–8]. Younger age, high baseline viral load (> 100,000 copies/mL) [2, 6] were factors found to be an independent predictors for virological discordance.

It is reported that people living with HIV with discordant responses were found to be at an increased risk to develop AIDS events such as opportunistic infections and severe non AIDS events such as stroke, liver and kidney failure, meningitis and endocarditis; risk factors for death [2, 8, 10, 14, 16, 17]. As a result, determination of immuno-virological discordant responses and associated risk factors among people living with HIV on HAART could have great role for policy makers and health care providers to end AIDS related epidemics by 2030 [18].

While immuno-virological response plays a key role to address treatment outcome, regimen change and management for people living with HIV using HAART, there is scant data in Ethiopia. To the best of our knowledge, there is a single study in Southern region of Ethiopia [5]. However, the study was done in small sample size (*n* = 86) and did not address factors associated with immunological and virological discordant responses. Moreover, this study cannot represent the national level prevalence of immunological and virological discordant. The limitations of the previous study calls for more data to be generated from each region with representative sample to forward reasonable findings and recommendations to help policy makers and implementers in order to plan and design proper intervention strategies to control HIV. The aim of this study was therefore, to fill the existing knowledge gap on the prevalence of immunological and virological discordant responses and associated predictors among people living with HIV on first line HAART in Tigray regional state, Northern Ethiopia.

## Methods

### Study area, setting and period

The study was conducted from September 1 to December 30, 2016 in Mekelle Hospital (MH) and Ayder comprehensive specialized hospital (ACSH), both located in Mekelle city. Mekelle, the capital city of Tigray Regional Sate is located 783 Km north of Addis Ababa, the capital city of Ethiopia. Of the five governmental hospitals found in Mekelle city, two hospitals namely MH and ACSH were selected purposely owing to their high patient flow. In 2016, around 8727 people were living with HIV (PLHIV) ever enrolled in MH, of these, 6789 were ever started HAART, and 4189 were actively on HAART. Similarly, in ACSH there were 1564 PLHIV ever enrolled of these, 1387 patients ever started with 1222 were actively on HAART.

### Study design and sampling technique

A cross sectional study was conducted to determine the immunological and virological discordant responses among people living with HIV who were taking first line HAART. The study participants were selected consecutively using convenient sampling technique.

### Study population and study variables

**Fig. 1:**
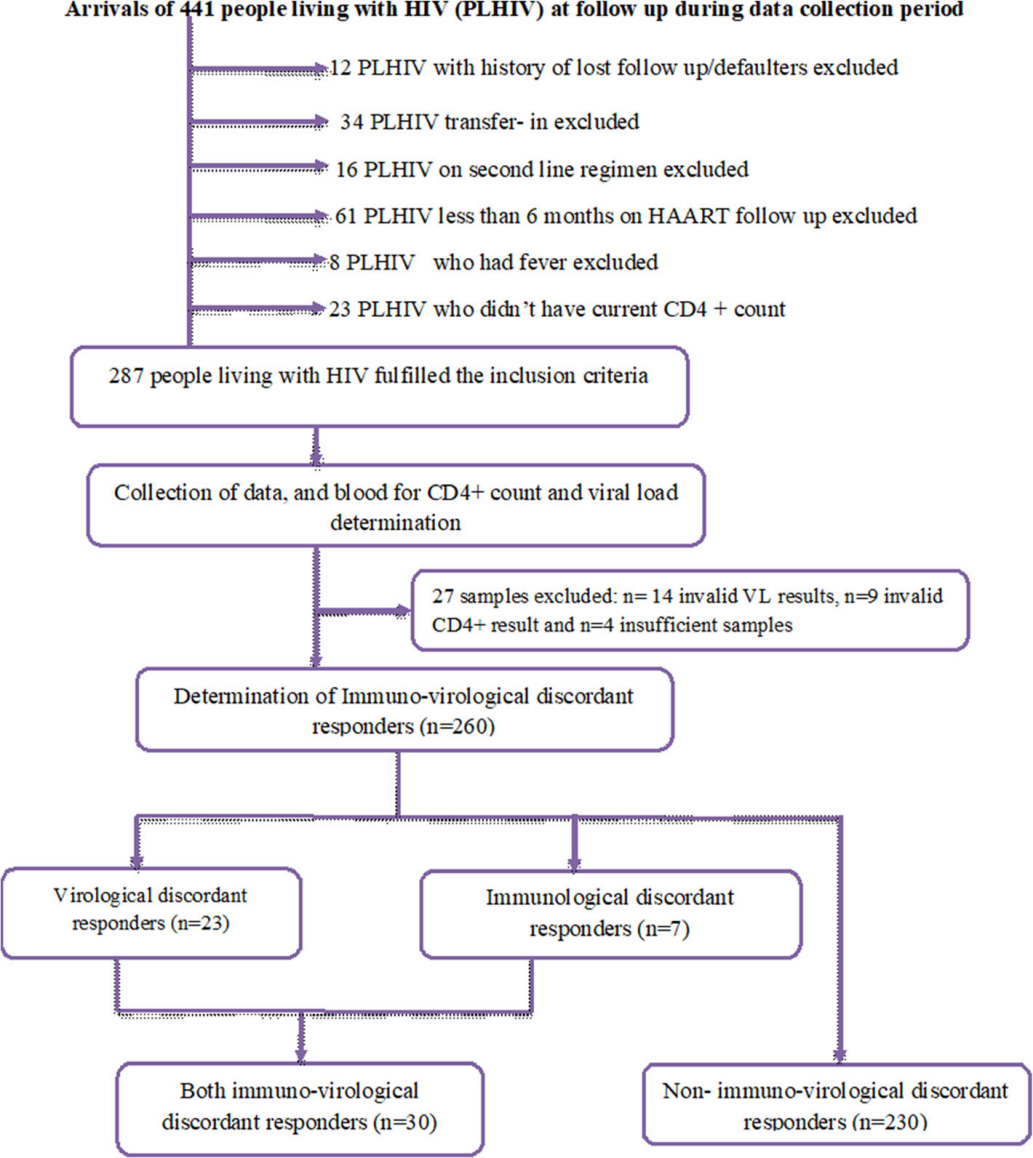
Work flow of the study

The study participants were patients 10 to 64 years of age who were on first line HAART for 7 months to 9 years. Patients who were less than 6 months on HAART follow up, who did not have current CD4+ count, who had been on second line regimen, lost to follow up, febrile, and transfer in individuals were excluded from the study. Of the total 441 people living with HIV who visited the hospitals during the study period, 154 were excluded as they did not fulfil the inclusion criteria, and 287 were included in the study (Fig. 1).

### Study variables

#### Outcome variables

Immunological and virological discordant responses.

#### Predictor variables

Socio-demographic characteristic, baseline and follow up clinical related data (such as WHO clinical stage, time on HAART, presence/absence of opportunistic infections, nutritional status, types of drugs and regimen given, HBV and HCV co-infections, level of adherence, and known chronic non communicable diseases) and laboratory data (CD4+ count and viral load).

### Data collection and laboratory testing methods

All people living with HIV who was on first line HAART for at least 6 months and visiting these health facilities during data collection period were included consecutively. All participants were requested for their willingness and included after written informed consent, and for minors consent from parents/guardians and assent from children. Baseline and follow-up clinical related data such as WHO clinical stage, type of regimen given, presence or absence of opportunistic infections, presence of chronic non-communicable diseases, nutritional status, HBV and HCV co-infections, any prophylaxis given, time interval from HIV diagnosis to HAART initiation), age at HAART initiation, and baseline and current (i.e. performed 3–4 months before data collection period) CD4+ count were collected from charts. In addition to recording socio demographic characteristics (such as age, sex, educational level, residence), blood samples for CD4+ count and viral load measurements were collected during the data collection time.

### Laboratory testing methods

#### Specimen collection and processing

Seven milliliter of blood was collected from each participant for CD4 + count and viral load test using vacutainer and poured in to two EDTA test tubes during data collection period. A test tube containing 3 mL of blood stored at room temperature was sent to ACSH ART laboratory for CD4+ count. The second test tube containing 4 mL of blood was centrifuged at 1600 rpm for 20 min using Sigma centrifuge (Sigma, serial number: 96853, Germany) in ACSH and the separated plasma was transported to Tigray Health Research Institute (THRI) using dry ice box and stored at − 80 °C until analyzed for viral load measurement.

#### RNA extraction and plasma viral load determination

HIV-1 RNA was extracted from 0.2 mL of plasma using Abbott *m2000sp* automated sample preparation system (Abbott Molecular, USA) in THRI according to manufacturer’s instructions during data collection period (at 36 months median time of HAART follow -up). Extracted RNAs were measured using Abbott *m2000rt* quantitative Real Time HIV-1 assay (Abbott Molecular, USA) with HIV-1 RNA detection level of 150 to 10 million copies/mL based on the manufacturer’s procedures.

#### CD4+ count

Baseline and current CD4+ count was collected from medical records. In addition, we collected blood samples from each participant and did last CD4+ count (to have persistent CD4+ count) and done at ACSH ART laboratory using the BD FACS Count™ (Becton Dickinson, USA) following the manufacturer’s protocol during data collection period (at 36 months median time of HAART follow up). Then, immunological failure was determined using the current and last (latest) CD4+ count.

### Definitions of terms

Virological failure: is defined as plasma viral load > 1000 copies/ml after at least 6 months of HAART initiation [19].

Viral suppression: Viral load level below < 150 copies/ml after at least 6 months of HAART initiation [19].

Immunological failure: defined as CD4+ count falls at or below250 cells/μl following clinical failure or persistent levels below100 cells/μl after 6 months of HAART initiation [19].

Clinical Failure: New or recurrent clinical event indicating severe immunodeficiency (clinical stage 4) after 6 months of HAART initiation [19].

Immunological discordant response: defined as plasma viral load level below 1000 copies/mL with immunological failure.

Virological discordant responses: defined as good immunological response with virological failure (i.e. single viral load >1000copies/mL).

Good immunological responses: defined as count achieving at, below or above 250 cells/μl without clinical failure or persistent CD4+ count above 100 cells/μl after 6 months of HAART.

Current CD4+ count: defined as CD4+ count result performed within 3–4 months interval before data collection period.

Adherence classification and definition: “good adherence” (missing ≤3 doses in a month, ≤ 95%), “fair adherence” (missing 4–8 doses in a month, 85–94%), and “poor adherence” (missing ≥9 doses in a month, < 85%) based on self-report and/or pill count [20].

### Data quality assurance, processing and analysis

To maintain quality, sample collection, handling, transportation, and laboratory work was done using the standard operational procedures. Moreover, the expiry date of reagents, completeness, and consistency of filled questionnaires was checked. We used Epidata version 3.1 to enter data and SPSS version 21 for statistical analyses. Median and interquartile ranges (IQR) were used to analyze continuous variables and counts with percentages for categorical variables. Fischer’s exact test was used to see statistical association of the variables with immunological discordant responses, whereas, binary and multiple logistic regressions were used to determine the associations of independent variables with virological discordant responses. Statistically significant was set at a *p*-value < 0.05 with 95% confidence interval.

## Results

### Characteristics of immuno-virological discordant respondents

Of the total of 287 participants included, 27 samples were excluded due to: invalid CD4+ results (*n* = 9), invalid viral load result (*n* = 15) and insufficient samples (*n* = 4). Hence we only included the data of 260 participants in the data analysis. From these, more than half (58.1%) of them were females. The median age at HAART initiation and at 36 months median time of HAART follow up was 35 (Interquartile range (IQR): 28–44) and 39 (IQR: 32–48), years, respectively. Similarly, median CD4+ count at 36 months median time of HAART follow-up was 384 (IQR: 239–572) cells/mm^3^. About 63% of the total participants had history of opportunistic infections at least once at baseline/follow up and 51% of them took isoniazid (INH) prophylaxis. Majority (74%) of them started HAART at 11 mean time interval (standard deviation (SD): + 19) of HIV diagnosis. Of the total 239 patients, who took Cotrimoxazole prophylaxis, 48% of them used for a median time of 30 months (IQR): 18–48) (Table 1).

**Table 1:** Characteristics of Immuno-virological responders among people living with HIV on HAART Mekelle hospital and Ayder Comprehensive Specialized Hospital (*n* = 260)

Characteristics	Immuno-virological Discordant Responses		Total (n %))
	No (n (%))	Yes (n (%))	
**Gender**			
Male	90 (82.6)	19 (17.4)	109 (100)
Female	140 (92.7)	11 (7.3)	151 (100)
**Age (years) at 36 months median time of HAART follow up**			
< 39	122 (86.5)	19 (13.5)	141 (100)
> 39	108 (90.8)	11 (9.2)	119 (100)
**Educational level**			
No education	54 (85.7)	9 (14.3)	63 (100)
Primary	66 (83.5)	13 (16.5)	79 (100)
Secondary and above	110 (93.2)	8 (6.8)	118 (100)
**Residence**			
Rural	41 (83.7)	8 (16.3)	49 (100)
Urban	189 (89.6)	22 (10.4)	211 (100)
**Chronic non-communicable diseases**^a^			
Yes	9 (75.0)	3 (25.0)	12 (100)
No	221 (89.1)	27 (10.9)	248 (100)
**INH Prophylaxis given**			
Yes	119 (93.0)	9 (7.0)	128 (100)
No	111 (84.1)	21 (15.9)	132 (100)
**History of TB co-infection**			
Yes	37 (86.0)	6 (14.0)	43 (100)
No	193 (88.9)	24 (11.1)	217 (100)
**History of opportunistic infections (other than TB)**^b^			
Yes	126 (88.1)	17 (11.9)	143 (100)
No	104 (88.9)	13 (11.1)	117 (100)
**Adherence to treatment**			
Fair/Poor	9 (50.0)	9 (50.0)	18 (100)
Good	221 (91.3)	21 (8.7)	242 (100)
**Regimen given**			
TDF based^c^	179 (90.9)	18 (9.1)	197 (100)
AZT based^d^	49 (81.7)	11 (18.3)	60 (100)
ABC based^e^	2 (66.7)	1 (33.3)	3 (100)
**Age (years) at HAART initiation**			
< 35	113 (85.6)	19 (14.4)	132 (100)
> 35	117 (91.4)	11 (8.6)	128 (100)
**WHO clinical stage at baseline**			
I/II	112 (91.1)	12 (8.9)	124 (100)
III/IV	118 (86.8)	18 (13.2)	136 (100)
**History of malnutrition**			
Yes	68 (81.9)	15 (18.1)	83 (100)
No	162 (91.5)	15 (8.5)	177 (100)
**Cotrimoxazole prophylaxis given (months) (*N*** **= 239)**			
< 30	96 (83.5)	19 (16.5)	115 (100)
> 30	113 (91.1)	11 (8.9)	124 (100)
**Viral load (copies/mL) at 36 months median time of HAART follow up**			
< 1000	221 (96.1)	9 (3.9)	230 (100)
> 1000	9 (30.0)	21 (70.0)	30 (100)
**CD4+ count (cells/μl) at baseline**			
< 100	67 (87.0)	10 (13.0)	77 (100)
101–200	69 (85.2)	12 (14.8)	81 (100)
201–300	49 (92.5)	4 (7.5)	53 (100)
> 300	45 (91.8)	4 (8.2)	49 (100)
**CD4+ count (cells/μl) at 36 months median time of HAART follow up**			
< 100	9 (56.2)	7 (43.8)	16 (100)
101–200	27 (79.4)	7 (20.6)	34 (100)
201–300	34 (81.0)	8 (19.0)	42 (100)
> 300	160 (95.2)	8 (4.8)	168 (100)
**Time interval from HIV Diagnosis to HAART initiation (months)**			
< 12	175 (87.1)	26 (12.9)	201 (100)
> 12	55 (93.2)	4 (6.8)	59 (100)
**HBV co- infection(*n*** **= 139)**			
Yes	7 (63.6)	4 (36.4)	11 (100)
No	114 (89.1)	14 (10.9)	128 (100)
**HCV co-infection(*n*** **= 64)**			
Yes	0 (0.0)	2 (100)	2 (100)
No	53 (85.5)	9 (14.5)	62 (100)

Note: *INH* Isoniazid, *TB* Tuberculosis, *HBV* Hepatitis B virus, *HCV* Hepatitis C virus. ^a^ Includes Hypertension and Diabetic mellitus. ^b^ Includes: oral/esophageal candidiasis; Pneumonias; Diarrhea; Zoster e.t.c ^c^ Drugs used concomitantly with TDF (Tenofovir) were: 3TC (lamivudine) + EFV (efaverinz) / NVP (Nevirapine); ^d^ Drugs used concomitantly with AZT (zidovudin); were: AZT+ 3TC+ NVP/ EFV; ^e^ Drugs used concomitantly with ABC (abacabir) were: 3TC and EFV

### Immuno-virological discordant responses and associated risk factors

A total of 30 (11.50% (95% CI = 11.46–11.54%)) patients had immuno-virological discordant responses. Among the discordant responders, 23/30 (76.7%) of them who had virological failure were immunologically recovered (*p* < 0.001) (data not shown). Study participants with no formal education were 4.47 times more likely to experience immuno-virological discordant responses ((AOR (95% CI) = 4.47(1.03–19.39, *p* = 0.045)) compared to these who attended secondary school and above. In addition, immuno-virological discordant responses were 80% less likely to occur in study participants who had viral load less than1000 copies/mL in a 36 median time of HAART initiation (AOR(95% CI) = 0.20 (0.05–0.86), *p* < 0.001). Immuno-virological discordant response were also statistically associated with HBV (*p* = 0.037) and HCV (*p* = 0.027) co-infections (Sup. Table 1).

### Immunological discordant responses and associated factors

Immunological discordance was found in 2.70% (95% CI = 2.68–2.72%) of the study participants. Though not statistical significant, about 57.1 and 71.4% of the immunologically discordant study participants were females and greater than 35 years age at HAART initiation, respectively (*p* > 0.05), and all were on TDF based regimen. More than half (57.1%) with immunologically discordant responses had history of TB co- infections (*p* = 0.016). At baseline, all of the patients were at WHO clinical stage III/IV and 71.4% of them had CD4+ count less than 100 cells/μl (*p* = 0.026) (data not shown).

About 18.2% of the HBV co-infected patients had immunological discordant response compared to mono-infected individuals (*p* = 0.05). In addition, 11.1% of the study participants with poor/fair treatment adherence experienced immunological discordant responses compare to their counter parts (*p* = 0.08) (Table 2).

**Table 2:** Immunological discordant responses and associated factors among HAART users in Mekelle Hospital and Ayder Comprehensive Specialized Hospital (*n* = 260)

Characteristics	Immunological discordance	***P***-value*
	Yes n (%)	
**Gender**		
Male	3 (2.8)	1.000
Female	4 (2.6)	
**Age (years) at HAART initiation**		
< 35	2 (2.4)	1.000
> 35	5 (2.8)	
**Age (years) at 36 months median time of HAART follow up**		
< 39	3 (2.1)	0.706
> 39	4 (3.4)	
**Residence**		
Rural	1 (2.0)	1.000
Urban	6 (2.8)	
**Chronic non-communicable diseases**^a^		
Yes	0 (0)	1.000
No	72.8)	
**TB co-infection**		
Yes	4 (9.3)	0.016
No	3 (1.4)	
**History of Opportunistic Infections (other than TB)**^b^		
Yes	3 (2.1)	0.704
No	4 (3.4)	
**HBV co-infection**		
Yes	2 (18.2)	0.05
No	3 (2.8)	
**HCV co-infection**		
Yes	1 (50.0)	0.062
No	1 (1.6)	
**Adherence to treatment**		
Poor/fair	2 (11.1)	0.08
Good	5 (2.1)	
**WHO clinical stage at baseline**		
I and II	0 (0.0)	
III and IV	7 (5.1)	
**CD4+ count (cell/μl) at baseline**		
< 100	5 (6.5)	0.026
> 100	2 (1.1)	
**Regimen given**		
TDF based^c^	7 (3.6)	
AZT based^d^	0 (0)	

Note: *P-value is determined by Fisher’s exact test, *INH* Isoniazid, *TB* Tuberculosis, *WHO* World Health Organization, *HBV* Hepatitis B virus, *HCV* Hepatitis C virus. ^a^ Includes Hypertension and Diabetic mellitus. ^b^ Includes: oral/esophageal candidiasis; Pneumonias; Diarrhea; Zoster e.t.c. ^c^ Drugs used concomitantly with TDF (Tenofovir) were: 3TC (lamivudine) + EFV (efaverinz) / NVP (Nevirapine). ^d ^Drugs used concomitantly with AZT (zidovudin); were: AZT+ 3TC+ NVP/ EFV

### Virological discordant response and associated risk factors

Virological discordant responses were seen in 8.80% (95% CI = 8.77–8.84%) of the study participants. Among these virologically discordant responders, 69.6% (16/23) and 56.5% (13/23) of them were males and aged less than or equal to 35 years old at HAART initiation, respectively. Furthermore, 69.6% (vs. 48.1%) of them had CD4+ count less than 384 cell/μl at 36 median time of HAART initiation. The majority (86.9%) with good virological responses were with good treatment adherence. However, 38.9% (vs 6.6%) of these with poor/ fair treatment adherence had virological discordant responses (*p* < 0.001) (data not shown).

Adjusting for age, adherence to treatment and type of regimen given, virological discordant response was 1.71 times more likely to occur in males (AOR ((95% CI) =1.71(1.13–1.10), *p* = 0.029) compared to females. Patients whose, HAART initiating at less than or equal to 35 years of age) were 4.25 times more likely to experience virological discordance ((AOR (95% CI) = 4.25(1.48–12.23), *p* = 0.007). Furthermore, the odds of virological discordant response was 70% less likely to occur among individuals who took TDF based regimen ((AOR (95% CI) =0.30 (0.10–0.88), *p* = 0.028) compared with these on AZT based. Poor/fair treatment adherence was also predictor for virological discordant responses ((AOR (95% CI) =0.12 (0.03–0.48), *p* = 0.003) (Table 3).

**Table 3:** Virological discordance and associated risk factors among HAART users in Mekelle Hospital and Ayder Comprehensive Specialized Hospital (*n* = 260)

Characteristics	Virological discordance	Binary logistic regression		Multiple logistic regression	
	Yes n (%))	***P***-value	COR(95% CI)	***P***-value	AOR (95% CI)
**Gender**					
Male	16 (14.7)	0.007	3.54 (1.40–8.93)	0.029	1.71 (1.13–10.10)
Female	7 (4.6)		Ref		Ref
**Age (years) at HAART initiation**					
< 35	13 (15.5)	0.012	3.04 (1.27–7.26)	0.007	4.25 (1.48–12.23)
> 35	10 (5.7)		Ref		Ref
**Age (years) at 36 months median time of HAART follow up**					
< 39	19 (13.5)	0.290	1.53 (0.70–3.36)		
< 39	11 (9.2)		Ref		
**Residence**					
Rural	7 (14.3)	0.143	2.03 (0.787–5.25)		
Urban	16 (7.6)		Ref		
**Chronic non-communicable diseases**^a^					
Yes	3 (25.0)	0.079*			
No	20 (8.1)				
**History of TB co-infection**					
Yes	2 (4.7)	0.388*			
No	21 (9.7)				
**Opportunistic infections (other than TB)**^b^					
Yes	14 (9.8)	0.663	1.30 (0.54–3.13)		
No	9 (7.7)		Ref		
**HBV co-infection (*n*** **= 139)**					
Yes	2 (18.2)	0.274*			
No	11 (8.6)				
**HCV co-infection (*n*** **= 64)**					
Yes	1 (50.0)	0.137*			
No	8 (12.9)				
**WHO clinical stages at baseline**					
I and II	12 (9.7)	0.653	1.22 (0.52–2.87)		
III and IV	11 (8.1)		Ref		
**History of malnutrition**					
Yes	10 (12.0)	0.217	1.73 (0.73–4.12)		
No	13 (7.3)		Ref		
**Regimen given (*n*** **= 257)**					
TDF based^c^	11 (5.6)	0.003	0.26 (0.11–0.64)	0.028	0.30 (0.10–0.88)
AZT based^d^	11 (18.3)		Ref		Ref
**CD4+ count (cell/μl) at baseline**					
< 100	5 (6.5)	0.390	0.64 (0.23–1.78)		
> 100	18 (9.8)		Ref		
**CD4+ count (cell/μl) at 36 months median time of HAART follow up**					
< 384	16 (12.3)	0.056	2.47 (0.98–6.21)	0.340	1.71 (0.57–5.11)
> 384	7 (5.4)		Ref		Ref
**Adherence to treatment**^e^					
Good	16 (6.6)	0.000	0.11 (0.04–0.33)	0.003	0.12 (0.030–0.48)
Fair/poor	7 (38.9)		Ref		Ref
**Educational level**					
No education	5 (7.9)	0.607	1.37 (0.42–4.50)		
Primary	11 (13.9)	0.063	2.57 (0.95–6.94)		
Secondary and above	7 (5.9)		Ref		

Note: *P-value is determined by Fisher’s exact test; *COR* Crude odds ratio, *AOR* Adjusted odds ratio, *TB* Tuberculosis, *HBV* Hepatitis B virus, *HCV* Hepatitis C virus; ^a^ Includes Hypertension and Diabetic mellitus. ^b^ Includes: oral/esophageal candidiasis; Pneumonias; Diarrhea; Zoster e.t.c. ^c^ Drugs used concomitantly with TDF (Tenofovir) were: 3TC (lamivudine) + EFV (efaverinz) / NVP (Nevirapine). ^d^ Drugs used concomitantly with AZT (zidovudin); were: AZT+ 3TC+ NVP/ EFV. ^e^ Drugs used concomitantly with ABC (abacabir) were: 3TC and EFV

## Discussion

This study described immunological and virological discordant responses and associated risk factors among persons on HAART at 36 months median time of HAART follow up. Generally, 11.5% of the study participants experienced immuno-virological discordant responses. Our finding were comparable with a study conducted in Brazil, 9% [21]. However, it was lower than the findings from Southern Ethiopia, 29% [5] and Nigeria, 33% discordancy [7]. This variation may be related with differences in the definition of immune-virological discordant responses. For example, in our study the outcome variables were defined based on the 2016 WHO guideline [19]. Immunological discordance was defined as drop of CD4+ count below 250 cells/μl following clinical failure, or persistent CD4+ count below 100 cells/μl despite viral suppression (viral load < 150 copies/mL), and virological discordance response was defined as virological failure (viral load level > 1000 copies/mL) with immunological success [19]. However, the above mentioned authors defined immunological discordance as “failure to increase CD4+ cell count by 50 cells/μl” [5] and “fall in CD4+ count below baseline levels despite viral suppression. In addition, virological discordance was also defined as viral load level above 400 copies/mL with immunological success [7].

In median time of 36 month of HAART follow up, immunological discordant response was found 2.70%. Our result was much lower than the studies conducted in Southern Ethiopia, 15.1–20.9% [5], Rwanda, 29% [6], Nigeria,16% [7], South Africa, 24% [11], Oman, 26.9% [12], India, 13.59 and 21.1% [9, 13] and Italy, 26.1% [10]. These differences may be related with definition of immunological discordance. For example, the aforementioned researchers defined it as “failure to increase count by 50 cells/μl despite viral suppression (viral load < 150 copies/mL) [5], an increase of < 100 cells/mm^3^ at 12 months compared to baseline in spite of full virological suppression (VL < 40 copies/mL) [6, 9], failure to increase CD4+ count by 50 cells/μl or fall in CD4+ count below baseline levels despite viral suppression (viral load level below 400 copies/mL) [7, 9, 11] and CD4+ count below 200 and 350 cells/μl despite viral suppression (viral load level < 50 copies/mL) [10, 12].

TB co-infection, HBV co-infection and CD4+ count below 100 cells/μl at baseline were factors associated with immunological discordant responses. Multicenter cohort study conducted in Germany indicated that individuals who developed TB had slow immune recovery despite viral suppression as compared with these mono-infected individuals [22]. Our study also showed significant difference among TB co-infected individuals (*p* = 0.016). This finding is also in line with a study conducted in South Korea [23]. This might be explained as TB infection impairs cellular immune responses through *M. tuberculosis* induced apoptosis of CD4+ cells which subsequently lead to depletion of CD4+ cells and results in poor immunological recovery despite viral suppression [24].

It was statistically significant that 18.2%of HBV co-infected individuals had immunological discordant response (*p* = 0.05) compared to non HBV co-infected individuals, 2.8%. This finding in lines with a study conducted in Switzerland [25] and China [26]. These studies reported that those HBV co-infected individuals had poor immunological recovery as compared with those mono-infected though the impact is not stated.

Immunological discordant responses was also associated with low CD4+ count (< 100 cells/μl) at baseline (*p* = 0.026). Association of baseline CD4+ count with immunological discordance was also reported by studies conducted in Nigeria [7], Oman [12], Canada [27], India [9], Thailand [14], Italy [10] and China [26]. This may be related with diminution of CD4+ cells before initiation of antiretroviral therapy [27], which may be slow to reconstitute with antiretroviral therapy.

Furthermore, the current study indicated that all those immunological discordant responses were on TDF based regimen. Though not statistically significant, other study from Oman [12] also indicated that these who were on AZT based regimen were less likely to have immunological discordant responses (*p* > 0.05). In other hand, impact of types of regimen on immunological discordance was also reported by a study conducted in Italy [10].

Virological discordant responses in this study were found 8.8% at 36 months median time of HAART follow up. Our finding was comparable to other studies conducted in Southern Ethiopia, 9.3–13.9% [5], Rwanda, 5% [6] and German, 11.9% [28]. Unlike to our result, higher virological discordance was reported in Nigeria, 17% [7]. This difference may be related with definition of virological discordant response, which was defined by these above mentioned authors as viral load level of ≥400 copies / mL with immunological recovery.

In this study, virological discordance was 1.71 times more likely to occur in males (*p* = 0.029) compared to females. This was in line with the study conducted in Rwanda which reported lower odds of virological discordant responses among females as compared with males [6]. The possible explanation for this could be due to low health seeking behavior such as poor treatment adherence of males as compared with females [29–31]. However, the association between gender and virological discordance needs further study.

Another variable which was found as an independent predicator of virological discordance was age at initiation of HAART. Accordingly, in a multiple logistic regressions, initiation of HAART at age of less than or equal to 35 years old was 4.25 times more to experience virological discordance (*p* = 0.007) compared to initiating above 35 years old. This current finding was also supported by the study conducted in Nigeria, where the odds of virological discordant responses was 1.58 times more likely to occur (*p* = 0.08) in these who initiated HAART at younger age (< 35 years old) [7].

Our study also indicated that study participants on TDF based regimens were 70% less likely to experience virological discordant responses (*p* = 0.028) compared with study participants on AZT based. This finding was also consistent with the study conducted in Nigeria where 89% were less likely to have virological discordant even though significant difference not observed [7].

Furthermore, it was statistically significant that odds of virological discordant responses was 88% less likely to occur among study participants with good treatment adherence (*p* = 0.003) compared with these with poor/fair treatment adherence. This was also supported from the study conducted in Nigeria (*p* = 0.001) [7] which indicated that odds of virological discordant response was higher in these people living with HIV with poor/fair treatment adherence.

The strength of this study was that this is the first study in the region which can give information on immuno-virological discordance response for the regional and national government and other stakeholders. However, our study had limitation. First due to budget constraints we did not perform HIV drug resistance test for these who had viral load level > 1000 copies/mL with immunological recovery. Second, we couldn’t do prospective cohort study to see the cause and effect of the associated factors with immuno-virological discordance response.

## Conclusions

Over all, immuno-virological discordance response was found in 11.5% in the study population at 36 months median time of HAART follow up. TB co-infection, HBV co-infection, low CD4+ count at baseline and WHO clinical stages III/IV were associated factors for immunological discordant responses. Whereas, virological discordant response was associated with age at HAART initiation, type of regimen given, male gender and poor/fair treatment adherence. Therefore, due attention should be given to the identified factors. Moreover, given evaluation of immuno-virological response is important to address patient treatment outcome, regimen change and patient management, we recommend that decision of patient treatment response should be done using trends of both viral load and CD4+ count concurrently.

## Supplementary Information

**Additional file 1: Supplement Table 1.** Immuno-virological discordance responses & associated risk factors among HAART users in Mekelle Hospital & Ayder comprehensive specialized hospital (*n*=260).

## Data Availability

The datasets used and analyzed during the current study are available from the corresponding author on reasonable request.

## Abbreviations

AIDS: Acquired Immunodeficiency Syndrome
HAART: Highly Active Antiretroviral Therapy
HBV: Hepatitis B Virus
HCV: Hepatitis C Virus
HIV: Human Immunodeficiency Virus
PLHIV: People living with HIV
WHO: World Health Organization
