# Supplementary material for: Immunological and virological discordance among people living with HIV on highly active antiretroviral therapy in Tigray, Northern Ethiopia
Source: BMC Infect Dis. 2021 Jun 12;21:561. doi: 10.1186/s12879-021-06206-4 (PMC8196496; doi:10.1186/s12879-021-06206-4)
Supplement: Supplementary file 1 — Additional file 1: Supplement Table 1. Immuno-virological discordance responses & associated risk factors among HAART users in Mekelle Hospital & Ayder comprehensive specialized hospital (n=260). [file 12879_2021_6206_MOESM1_ESM.docx]

**Supplement Table 1**: Immuno-virological discordance responses & associated risk factors among HAART users in Mekelle Hospital & Ayder comprehensive specialized hospital (n=260).

| **Characteristics** | **Immuno-virological discordant** | **Binary logistic regression** | | **Multiple logistic regression** | |
| --- | --- | --- | --- | --- | --- |
|  | **Yes (N (%))** | **P-value** | **COR(95% CI)** | **P-value** | **AOR(95% CI)** |
| Gender | | | | | |
| Male | 19(17.4) | 0.014 | 2.69(1.22-5.91 | 0.257 | 1.97(0.61-6.34) |
| Female | 11(7.4) |  | Ref |  | Ref |
| Age(years) at HAART initiation | | | | | |
| < 35 | 15(17.9) | 0.031 | 2.33(1.08-5.04) | 0.08 | 3.04(0.89-10.43) |
| >35 | 15(8.5) |  | Ref |  | Ref |
| Age(years) at median time of 36 months of HAART follow up | | | | | |
| < 39 | 19(13.5) | 0.290 | 1.53(0.70-3.36) |  |  |
| >39 | 11(9.2) |  | Ref |  |  |
| Residence | | | | | |
| Rural | 8(16.3) | 0.248 | 1.68(0.70-4.03) |  |  |
| Urban | 22(10.4) |  | Ref |  |  |
| Chronic non-communicable diseases^1^ | | | | | |
| Yes | 3(25.0) | 0.149* |  |  |  |
| No | 27(10.9) |  |  |  |  |
| History of TB co-infections | | | | | |
| Yes | 6 | 0.588 | 1.30(0.50-3.41) |  |  |
| No | 24 |  | Ref |  |  |
| History of Opportunistic Infections(other than TB)^2^ | | | | | |
| No | 13(11.1) | 0.845 | 1.08(0.51-2.33) |  |  |
| Yes | 17(11.9) |  | Ref |  |  |
| WHO clinical stage at base line | | | | | |
| I/II | 12(9.7)) | 0.370 | 0.48(0.21-1.11) |  |  |
| III/IV | 18(13.2) |  | Ref |  |  |
| HBV co-infection | | | | | |
| Yes | 4(36.4) | 0.037* |  |  |  |
| No | 14(10.9) |  |  |  |  |
| Types of regimen |  |  |  |  |  |
| TDF based^3^ | 18(9.1) | 0.053 | 0.45(0.20-1.01) | 0.69 | 2.42(0.051-11.57) |
| AZT based^4^ | 11(18.3) |  | Ref |  | Ref |
| History of Malnutrition | | | | | |
| Yes | 15(18.1) | 0.027 | 2.38(1.10-5.14) | 0.48 | 1.53(0.47-4.97) |
| No | 15(8.5) |  | Ref |  | Ref |
| Adherence to treatment | | | | | |
| Fair/poor | 9(50.0) | 0.000 | 10.5(3.77-29.38) | 0.581 | 1.58(0.31-8.10) |
| Good | 21(8.7) |  | Ref |  | Ref |
| Viral load(copies/mL) at 36 median time of HAART | | | | | |
| <1000 | 9(3.9) | 0.000 | 0.17 (0.06-0.49) | 0.000 | 0.20(0.05-0.86) |
| >1000 | 21(70.0) |  | Ref |  | Ref |
| Educational level | | | | | |
| No education | 9(14.3) | 0.16 | 2.29(0.84-6.27) | 0.045 | 4.47(1.03-19.39) |
| Primary | 13(16.5) | 0.036 | 2.71(1.07-6.88) | 0.088 | 3.30(.84-13.00) |
| Secondary & above | 8(6.8) |  | Ref |  | Ref |
| HCV co-infection | | | | | |
| Yes | 2(100) | 0.027* |  |  |  |
| No | 9(14.5) |  |  |  |  |
| CD4 count(cells/μL) at base line | | | | | |
| < 100 | 10(13.0) | 0.636 | 1.22(0.54-2.74) |  |  |
| >100 | 20(10.9) |  | Ref |  |  |
| CD4 + count(cells/ μL) at 36 median time of HAART follow up | | | | | |
| < 384 | 23(17.7) | 0.003 | 3.78(1.56-9.15) | 0.123 | 2.69(0.76-9.44) |
| >384 | 5(5.4) |  | Ref |  | Ref |

**Note**: *P-value is determined by Fisher’s exact test; COR: crude odds ratio; AOR: Adjusted odds ratio; HAART: Highly active antiretroviral therapy; WHO: World Health Organization; HBV: Hepatitis B virus; HCV: Hepatitis C virus; **^1^** Includes Hypertension and Diabetic mellitus. ^2^ Includes: oral/esophageal candidiasis; Pneumonias; Diarrhea; Zoster e.t.c ^3^Drugs used together with TDF (Tenofovir) were: 3TC (lamivudine) EFV (efaverinz) (68.8 % of the study participants) & 3TC NVP (Nevirapine) (6.9 %); ^4^ Drugs used together with AZT (zidovudin); were: AZT 3TC NVP (16.2%) & AZT 3TC EFV (6.9%);
